# The H2BG53D oncohistone directly upregulates *ANXA3* transcription and enhances cell migration in pancreatic ductal adenocarcinoma

**DOI:** 10.1038/s41392-020-00219-2

**Published:** 2020-06-30

**Authors:** Yi Ching Esther Wan, Jiaxian Liu, Lina Zhu, Tze Zhen Evangeline Kang, Xiaoxuan Zhu, John Lis, Toyotaka Ishibashi, Charles G. Danko, Xin Wang, Kui Ming Chan

**Affiliations:** 1 Department of Biomedical Sciences, City University of Hong Kong, Hong Kong, China; 2 Key Laboratory of Biochip Technology, Biotech and Health Centre, Shenzhen Research Institute of City University of Hong Kong, Shenzhen, China; 3 Department of Molecular Biology and Genetics, Cornell University, New York, NY USA; 4 Division of Life Science, Hong Kong University of Science and Technology, Hong Kong, China; 5 USA James A Baker Institute for animal health, Cornell University, New York, NY USA

**Keywords:** Cancer genetics, Epigenetics

**Dear Editor,**

Histones are essential proteins in compacting genomic DNA and regulating gene expression. Previous studies on histone H3 oncohistones in pediatric brain cancers^1,2^ and chondroblastoma^3^, documented the transcriptomic reprogramming through the alterations of histone modifications. We recently reported the identification of a novel cancer associated mutation, the H2BG53-to-D in pancreatic ductal adenocarcinoma (PDAC)^4^. We showed that the H2BG53D mutation weakens the interaction between nucleosomal DNA and histone octamer, subsequently enhances transcription in vitro. We further showed that cells expressing the G53D mutant H2B acquired oncogenic phenotypes in our CRISPR-Cas9 knock-in model. However, the mechanism by which H2BG53D mutation promotes PDAC remains unknown.

To examine the effect of the H2BG53D mutation on gene expression we conducted RNA-seq using the CRISPR knock-in H2BG53D mutant and isogenic wild-type clones^4^. We identified 280 upregulated and 262 downregulated genes (Fig. 1a) which are associated with growth factor signaling, cell adhesion, and pathways involved in cancer development (Fig. 1b). Since G53D mutation weakens the DNA–histone interaction, we postulated that the increase in gene expression is a consequence of elevated transcriptional activity at genes containing G53D mutant H2B. We mapped active RNA polymerases by precision nuclear run-on sequencing (PRO-seq) and identified 559 genes with elevated PRO-seq signals and 426 genes with reduced signals (Fig. 1c). On a genome-wide scale, we observed significant correlation between differential gene expression and Pol II occupancy alteration between H2BG53D mutant and wild-type cells (Fig. 1d). Gene set enrichment analyses (GSEA) revealed that genes with significant upregulation have a remarkable increase in Pol II occupancy in PRO-seq (Supplementary Fig. 1a) and vice versa (Supplementary Fig. 1b).

**Fig. 1:**
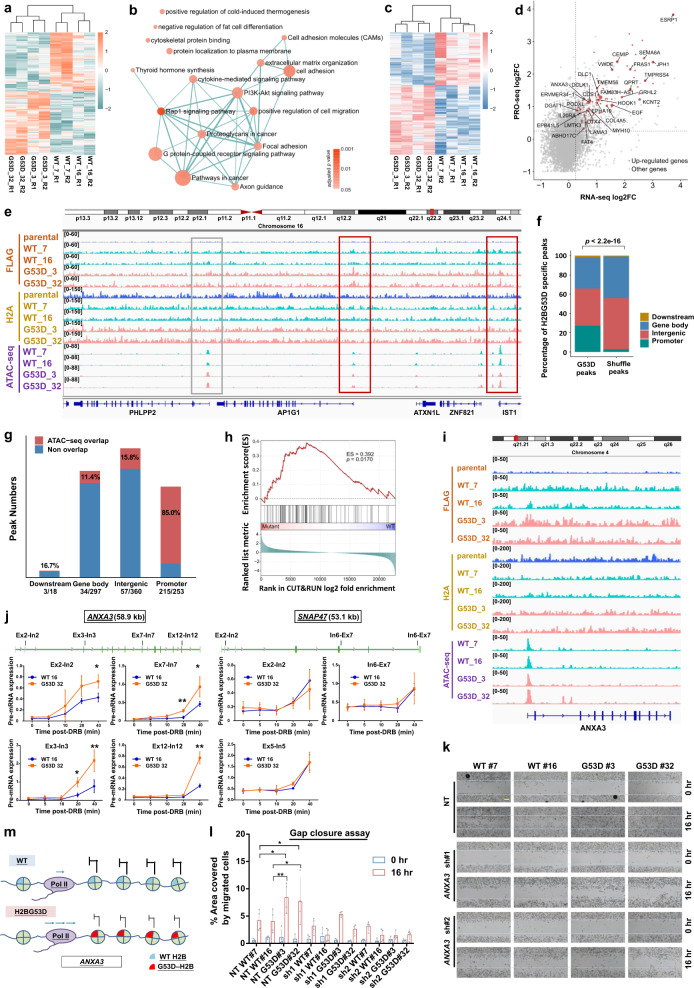
**a** Heat map showing the differential expression of genes in two wild-type and two H2BG53D mutant clones with two repeats. **b** Enrichment map of gene sets significantly overrepresented for differentially expressed genes between H2BG53D mutant and wild-type clones (Benjamini–Hochberg adjusted *p* < 0.05, hypergeometric tests). **c** Heat map showing the gene loci with differential Pol II occupancy. **d** Scatter plot illustrating the significant genome-wide correlation between RNA-seq and PRO-seq (Pearson correlation coefficient: 0.37, *p* < 2.2e−16). Ninety-nine genes with significantly elevated signals in both RNA-seq and PRO-seq are highlighted in red, among which 31 genes with significant FLAG enrichment in mutants are labeled with gene symbols and sized by the level of FLAG enrichment. **e** Genome viewer representations of normalized FLAG CUT&RUN reads for wild type and G53D mutant H2B (brown), H2A (yellow), and ATAC-seq reads (purple) in two wild-type (WT#7 & #16) and two H2BG53D mutant clones (G53D#3 & #32). Gray box represents area with ATAC-seq signal but without G53D-specific peak. Red boxes illustrate the overlapping of ATAC-seq and G53D-specific peaks. **f** Genomic distributions of the FLAG CUT&RUN H2BG53D-specific peaks (982) and randomly shuffled peaks (*p* < 2.2e−16, *χ*^2^ test). **g** Co-localization of H2BG53D-specific peaks with ATAC-seq peaks. A large proportion of promoter enriched H2BG53D peaks are co-localized with ATAC-seq peaks. **h** Gene set enrichment plot illustrating that the 99 genes with both significantly elevated RNA-seq and PRO-seq signals show significantly higher FLAG signal in G53D mutant than wild-type clones (*p* = 0.0170, permutation test, *n* = 10,000). The upper panel illustrates the running sum scores of GSEA (gene set enrichment analysis) random walks, the middle and lower panels show the positions of the 99 genes in the gene list ranked by FLAG log2-fold enrichment. **i** Genome viewer representations of normalized FLAG CUT&RUN reads for wild-type and G53D mutant H2B (brown), H2A (yellow), and ATAC-seq reads (purple) at the *ANXA3* gene locus in two wild type (WT#7 & #16) and two H2BG53D mutant clones (G53D#3 & #32). **j** Elevated transcription of *ANXA3* but not *SNAP47* in H2BG53D mutant cells. Levels of transcription of the indicated genes were detected by RT-qPCR using primers at indicated intro–exon boundaries (*p < 0.05, **p < 0.01). **k** shRNA knockdown of *ANXA3* reduced gap closure property. One representative set of data including the gap closure assay. RT-qPCR, western blotting (Supplementary Fig. 4) is shown from at least four independent repeats. Scale bar, 200 µm. **l** Quantification of four independent gap closure assays by Image J (*p < 0.05, **p < 0.01), error bar showing standard deviation. **m** A model for the effect of H2BG53D mutation on transcriptional regulation of *ANXA3*

Compared to wild-type cells, a total of 99 genes showed significantly elevated signals in both RNA-seq and PRO-seq in mutant cells (Fig. 1d and Supplementary Table 1). This group of genes is of interest because their upregulation is likely accountable to changes on the transcription level. On the other hand, increased mRNA level without changes in Pol II occupancy (181 genes), or vice versa (460 genes), indicate the involvement of post-transcriptional control (Supplementary Fig. 1c).

To determine the direct effect of the H2BG53D mutation on gene regulation, we mapped the genomic localization of the G53D-H2B in CRISPR knock-in cell lines^4^ by FLAG CUT&RUN assays while using H2A CUT&RUN as internal control (Fig. 1e). We identified 928 G53D-specific peaks by differential occupancy analysis compared to wild-type cells, 253 of which reside in the promoter region, showing a significant difference (*p* < 2.2e−16) when compared with the randomly shuffled peaks with the same length of G53D peaks (Fig. 1f). A significant number of the promoter-bound G53D peaks co-localized with open chromatin regions (Fig. 1g) as revealed by ATAC-seq (Fig. 1e), suggesting that these G53D-H2B enriched regions might impact gene transcription. However, the majority of the G53D peaks at intergenic region and gene bodies are not located at open chromatin regions, indicating that the G53D mutant H2B might have additional effects on the chromatin. When correlating the genomic distribution of G53D peaks (Fig. 1e–g) to gene expression changes (Fig. 1a–d), we found that only a small proportion of the genes with promoter enrichment of G53D had increased gene expression (3 out of 253), suggesting that the promoter-G53D enrichment alone is insufficient to influence transcription. To elucidate whether locus-wide enrichment of G53D-H2B is required for altering transcription in vivo, we identified genes showing differential occupancy of FLAG-tagged histones between wild-type and H2BG53D mutant cells from 1 kb upstream of TSS to TES. GESA analysis showed that the 99 genes with elevated gene expression (detected by RNA-seq) and transcription (detected by PRO-seq) (Supplementary Fig. 1) positively correlated with H2BG53D enrichment (Fig. 1h), suggesting that the G53D mutant H2B directly promotes transcription of these targets in vivo. More specifically, 31 genes show locus-wide differential enrichment of G53D-H2B, indicating that the locus-wide, rather than local enrichment of G53D-H2B at promoter, impacts target gene transcription (Fig. 1h and Supplementary Table 1). Gene ontology analysis of the RNA-seq data showed that genes involved in pathways related to cell migration, such as cell adhesion and focal adhesion, are differentially expressed in the H2BG53D mutant cells (Fig. 1b), suggesting that the H2BG53D mutation might target cell migration related phenotypes in PDAC. For this reason, among the 31 candidates we focused on the genes that are involved in cell migration and cancer metastasis. We found that *ANXA3*, an oncogene that has been shown to play important roles in metastasis, is overexpressed in lung, liver^5^ and ovarian carcinomas. However, the functional significance and the clinical relevance of *ANXA3* in PDAC remain elusive. Hence, we selected *ANXA3* and investigated its function in PDAC in the context of the H2BG53D mutation.

The G53D-H2B occupancy at *ANXA3* was revealed by FLAG CUT&RUN (Fig. 1i and Supplementary Fig. 2a) and confirmed by independent CUT&RUN assays (Supplementary Fig. 2b) as well as MNase FLAG ChIP-qPCR (Supplementary Fig. S2c). The elevated expression of *ANXA3* (Supplementary Fig. 3a) was confirmed by RT-qPCR and western blot (Supplementary Fig. 3b, c). To investigate the direct effect of the G53D mutant H2B on *ANXA3* transcription in vivo, H2BG53D cells were treated with 5,6-dichloro-1-beta-ribofuranosyl benzimidazole (DRB) to arrest transcription elongation by RNA Pol II (Fig. 1j and Supplementary Fig. 3d). By RT-qPCR we found that the level of *ANXA3* primary transcript was reduced significantly in both wildtypes and H2BG53D mutants upon DRB treatment, but gradually increased after released from DRB inhibition (Fig. 1j and Supplementary Fig. 3d). Importantly, substantially more primary *ANXA3* transcripts were found at all tested exon–intron junctions along the gene in time course experiments in the two H2BG53D clones, indicating increased transcription elongation on *ANXA3*. Conversely, *SNAP47*, a gene that does not have specific FLAG-H2BG53D enrichment, showed comparable recovery after DRB treatment. Together, these results suggested that the H2BG53D plays a direct role in elevating the transcription of *ANXA3*.

We performed oncogenic assays and found that depletion of *ANXA3* by shRNAs reduced the cell migration abilities in both wild-type and H2BG53D clones, when compared with their respective non-targeting controls (Fig. 1k, l and Supplementary Fig. 4). However, the H2BG53D mutant cells still exhibited faster migration than wild-type cells, suggesting that other genes or pathways also contribute to the increased oncogenic properties in the H2BG53D cells. More importantly, we found that PDAC patients with high *ANXA3* expression had poor overall survival (Supplementary Fig. 5a). We also observed that patients harboring the H2BG53D mutation have higher *ANXA3* expression (Supplementary Fig. 5b) and poorer prognosis compared to non-H2BG53D patients (Supplementary Fig. 5c), although these are not statistically significant which is likely due to the limited sample size. These findings further support the clinical relevance of the H2BG53D mutation in PDAC.

Together with the findings reported earlier by us^4^, we propose a model in which the H2BG53D mutation weakens the interaction between nucleosomal DNA and histone octamer, elevates the expression of cancer associated genes and leads to enhanced oncogenic properties in PDAC (Fig. 1m). Our work highlights the clinical significance of the H2BG53D mutation in PDAC and provides the basis of developing new strategies in treating patients harboring this histone mutation.

## Supplementary information

Supplementary Information

## Data Availability

RNA-seq, PRO-seq, ATAC-seq, and CUT&RUN sequencing data sets have been deposited to the Gene Expression Omnibus under accession number GSE134864.

## References

[1] Chan KM (2013). A lesson learned from the H3.3K27M mutation found in pediatric glioma A new approach to the study of the function of histone modifications in vivo?. Cell Cycle.

[2] Chan K-m (2013). The histone H3.3K27M mutation in pediatric glioma reprograms H3K27 methylation and gene expression. Genes Dev..

[3] Fang D (2016). The histone H3. 3K36M mutation reprograms the epigenome of chondroblastomas. Science.

[4] Wan YCE (2020). Cancer-associated histone mutation H2BG53D disrupts DNA–histone octamer interaction and promotes oncogenic phenotypes. Signal Transduct. Target. Ther..

[5] Tong M (2015). ANXA3/JNK signaling promotes self-renewal and tumor growth, and its blockade provides a therapeutic target for hepatocellular carcinoma. Stem Cell Rep..

