# Supplementary material for: The H2BG53D oncohistone directly upregulates ANXA3 transcription and enhances cell migration in pancreatic ductal adenocarcinoma
Source: Signal Transduct Target Ther. 2020 Jun 30;5:106. doi: 10.1038/s41392-020-00219-2 (PMC7326972; doi:10.1038/s41392-020-00219-2)
Supplement: Supplementary file 1 — Supplementary Information [file 41392_2020_219_MOESM1_ESM.pdf]

Supplementary data - figures and tables

The H2BG53D oncohistone directly upregulates *ANXA3* transcription and enhances cell migration in Pancreatic Ductal Adenocarcinoma

Yi Ching Esther Wan[1,2#], Jiaxian Liu[1,2#], Lina Zhu[1,2#], Tze Zhen Evangeline Kang[1,2], Xiaoxuan Zhu[1,2], John Lis[3], Toyotaka Ishibashi[4], Charles G. Danko[5*], Xin Wang[1,2*], Kui Ming Chan[1,2*]

[1]Department of Biomedical Sciences, City University of Hong Kong, Hong Kong, China

[2]Key Laboratory of Biochip Technology, Biotech and Health Centre, Shenzhen Research Institute of City University of Hong Kong, Shenzhen, China

[3]Department of Molecular Biology and Genetics, Cornell University, NY, USA

[4]Division of Life Science, Hong Kong University of Science and Technology, Hong Kong, China

[5]USA James A Baker Institute for animal health, Cornell University, NY, USA

This supplementary data file contains:

- Supplementary Figure 1-5
- Supplementary Table 1-2
- Supplementary Methods
- References

Supplementary Fig.1

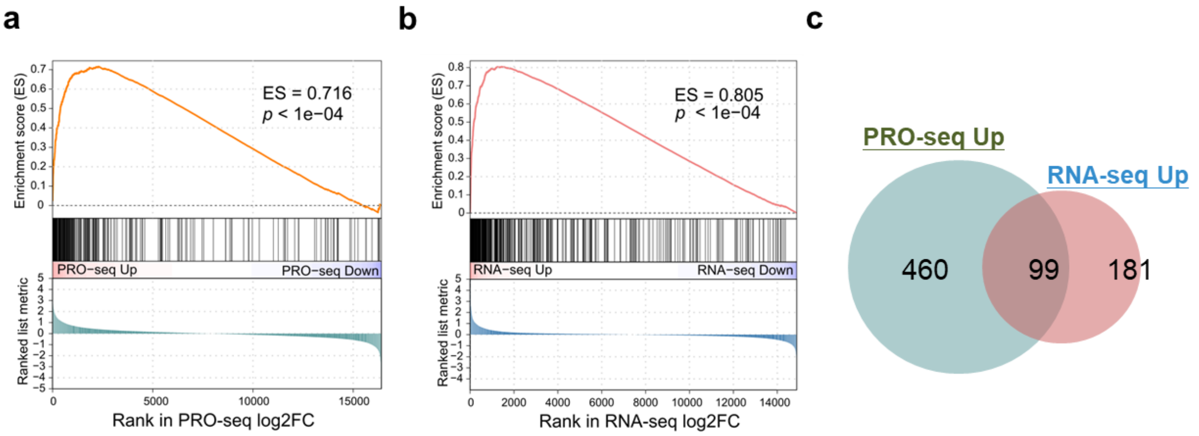

Supplementary Fig.1 | Differential gene expression and Pol II occupancy alteration between H2BG53D mutant and wildtype cells are well correlated. (a) Gene set enrichment plot shows genes with significantly elevated RNA-seq signals have remarkably increased Pol II occupancy in PRO-seq (p < 1e-04, permutation test, n=10,000). The upper panel illustrates the running sum scores of GSEA (Gene Set Enrichment Analysis) random walks, the middle and lower panels show the positions of genes with significantly elevated RNA-seq signals in the gene list ranked by PRO-seq log2 fold change. **(b)** Gene set enrichment plot shows genes with elevated PRO-seq signals have significantly higher expression in RNA-seq (p < 1e-04, permutation test, n=10,000). The middle and lower panels show the positions of genes with significantly elevated PRO-seq signals in the gene list ranked by RNA-seq log2 fold change. **(c)** Venn diagram illustrating that 99 genes have elevated signals in both RNA-seq and PRO-seq.

Supplementary Fig.2

a

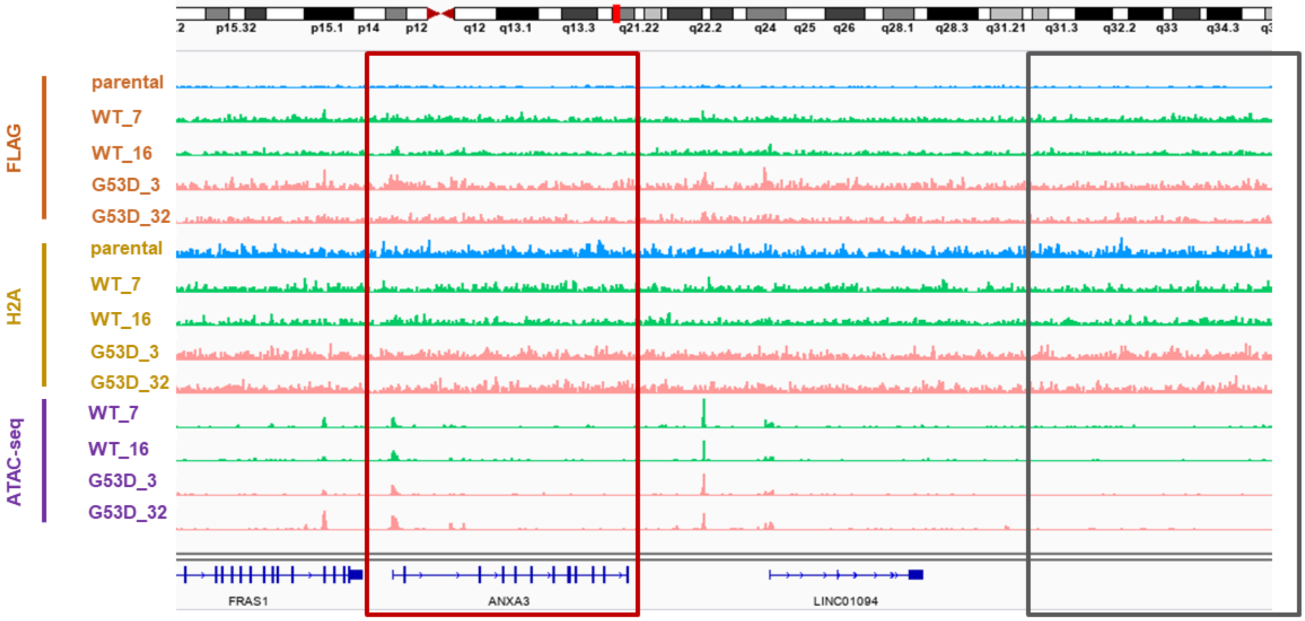

b

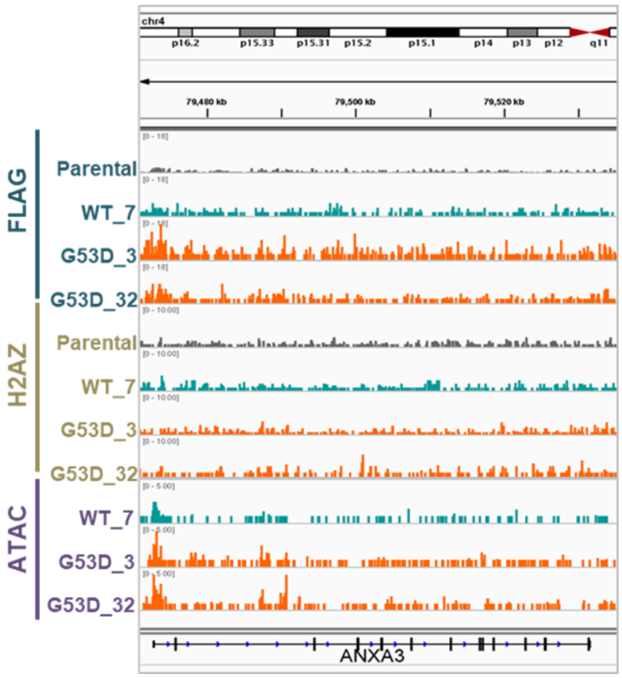

c

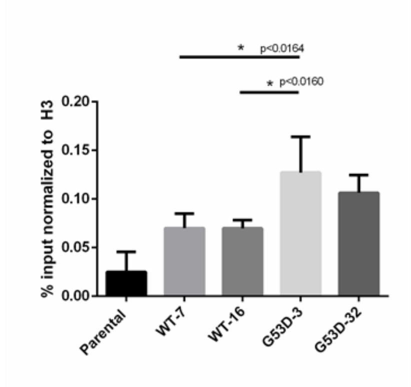

Supplementary Fig.2 | H2BG53D is enriched at *ANXA3*. (a) Genome viewer showing region with H2BG53D-FLAG enrichment (*ANXA3*, red box), and a region without specific H2BG53D-FLAG enrichment (downstream of *LINC01094*, black box). **(b)** Validation of H2BG53D enrichment at *ANXA3* locus by independent FLAG CUT&RUN in wildtype and two H2BG53D mutant lines. Parental cells served as negative control for the FLAG CUT&RUN experiments. **(c)** qPCR amplicon centers +608 (relative to TSS) of the *ANXA3* locus. Error bars indicate standard deviation from three independent experiments.

Supplementary Fig.3

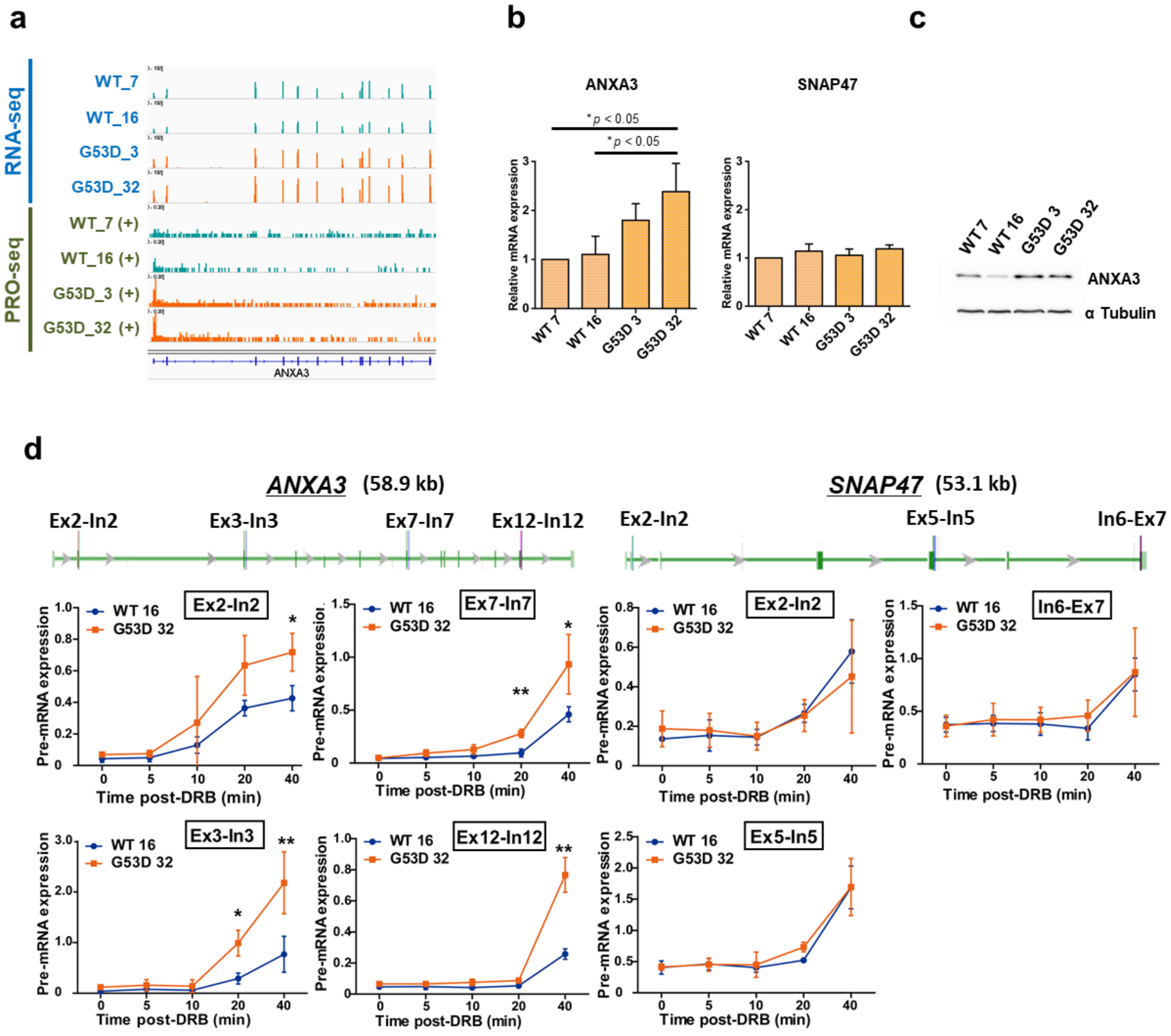

Supplementary Fig.3 | H2BG53D alters transcription of *ANXA3 in vivo*. (a) Genome viewer showing the normalized RNA-seq reads (upper) and PRO-seq reads (lower) of ANXA3 in two wildtype and two H2BG53D cell lines. Validation of the elevated expression of *ANXA3* by **(b)** RT-qPCR (*$p < 0.05$; LSD post hoc one-way ANOVA test) and **(c)** Western Blotting. **(d)** Levels of transcription of the indicated genes were detected by RT-qPCR using primers at indicated intron-exon boundaries. (* $p < 0.05$) Schematic diagram showing exon (Ex)–intron (In) junctions along the gene body of *ANXA3* and *SNAP47*. Cells were first incubated with 300 μM of DRB for 3.5 hours and then the cells were washed with PBS and further incubated in fresh medium for the indicated times. Levels of pre-mRNA of the regions were measured by RT-qPCR. Pre-mRNA values are normalized to the values of DMSO-treatment control, which was set to 1. Results are shown as means ± standard deviation (SD) from three independent experiments (*$p < 0.05$ vs. WT with unpaired *t*-test).

Supplementary Fig.4

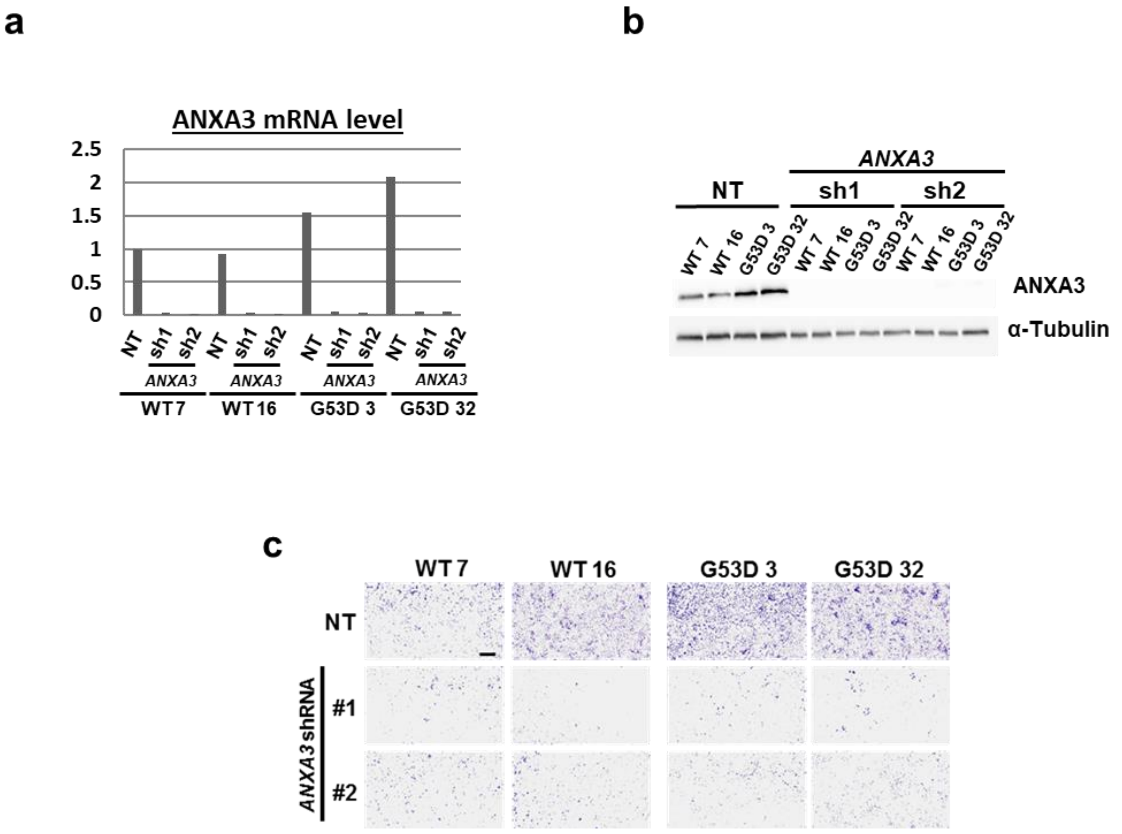

Supplementary Fig.4 | ANXA3 depletion in H2BG53D cell lines reverse migration properties. Depletion of ANXA3 by shRNA (knockdown efficiency measured by **(a)** RT-qPCR and **(b)** western blot) reduced the migration ability in both wildtype and G53D cell lines. Migration ability was measure by both gap closure assay (Figure 1k) and transwell migration assay **(c),** showing representative image from 3 independent experiments**.**

Supplementary Fig.5

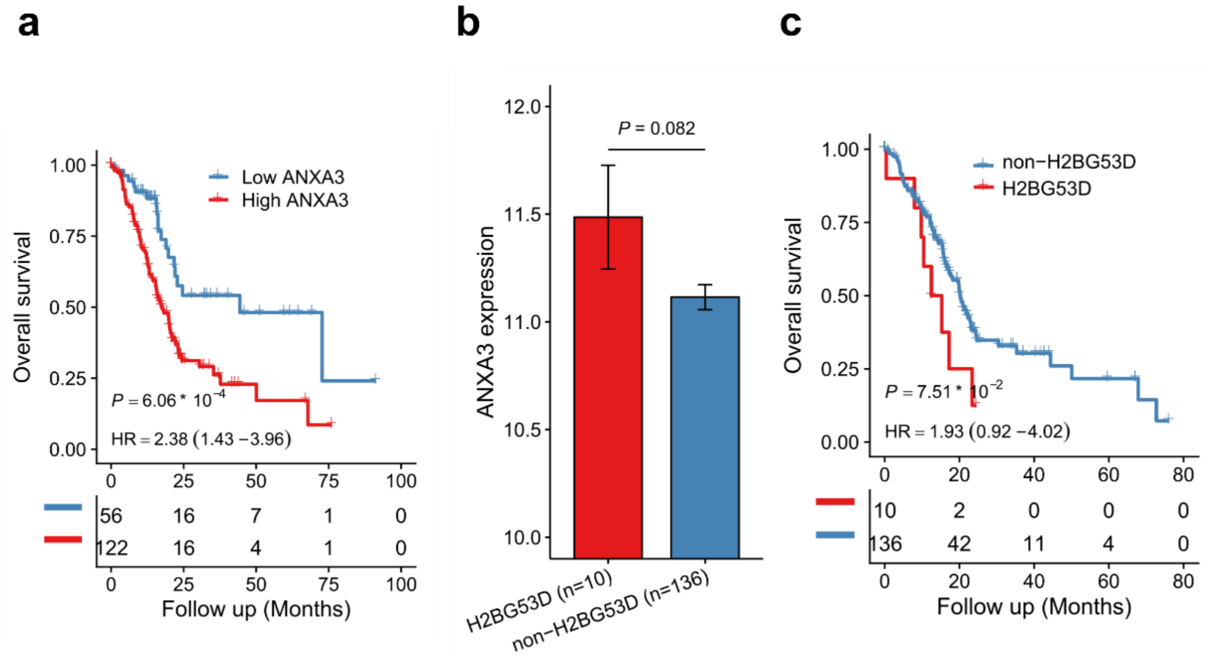

Supplementary Fig.5 | H2BG53D is correlated with higher *ANXA3* expression and poor survival. (a) Gene expression analysis of *ANXA3* of the 146 PDAC patients available from the TCGA database. The 10 H2BG53D patients have higher *ANXA3* expression compared to the 136 non-H2BG53D PDAC patients. p=0.082, one-tailed, two-sample t-test. **(b)** Comparison of the overall survival between PDAC patients with (10 patients) and without (136 patients) the H2BG53D mutation in TCGA data set. p=0.075, log-rank test. **(c)** Comparison of the overall survival between PDAC patients with high *ANXA3* expression and low *ANXA3* expression in TCGA data set (divided based on the mean expression of *ANXA3*). p=6.06e^-4, log-rank test.

Supplementary Table 1

The 99 genes showing significant elevated signals in both RNA-seq and PRO-seq in H2BG53D mutant cells.

Gene	RNA-seq log2FC	RNA-seq *p*	PRO-seq log2FC	PRO-seq *p*	CUT&RUN log2FE	CUT&RUN *p*
EGF	1.56	0.0143	1.05	0.0364	0.717	0.000436
DGAT1	0.326	0.0268	0.87	0.0319	0.814	0.000912
CEMIP	1.74	8.25E-11	2.38	3.55E-05	0.632	0.00107
FAT4	0.598	0.0145	0.521	0.00606	0.609	0.00129
JPH1	3.03	6.29E-16	2.37	5.52E-05	0.63	0.00135
FAM83H-AS1	1.15	0.0016	1.29	0.00204	0.834	0.00138
EPHA10	1.02	0.0364	1.12	0.00348	0.625	0.00161
KCNT2	2.55	0.0469	1.22	0.0148	0.615	0.00174
ABHD17C	0.551	1.68E-07	0.338	0.0442	0.627	0.00181
TMPRSS4	2.74	0.0151	1.8	0.0297	0.624	0.00198
LAMA3	0.552	0.0107	0.733	0.00479	0.527	0.00214
GRHL2	1.91	0.0162	1.29	0.0045	0.558	0.00268
CDS1	0.775	0.0326	1.17	0.00658	0.611	0.00332
FRAS1	2.21	9.85E-08	2.15	0.000199	0.594	0.00379
IL20RA	0.67	0.0176	0.884	0.00112	0.602	0.00399
ESRP1	3.75	0.00052	3.83	8.60E-07	0.615	0.00431
HOOK1	1.05	5.98E-08	1.21	3.56E-07	0.562	0.00433
EPB41L5	0.416	9.39E-05	0.805	0.000597	0.541	0.00443
LMTK3	0.473	0.00464	0.581	0.0417	0.702	0.00468
DLC1	1.07	5.73E-05	1.36	3.36E-06	0.603	0.00483
ERVMER34-1	0.726	0.00904	1.14	0.0424	0.78	0.00515
MYH10	0.724	0.00125	0.942	0.0271	0.581	0.00521
ANXA3	0.566	0.0221	1.24	0.00147	0.61	0.00576
DCLK1	0.749	0.0189	1.17	0.000247	0.523	0.00627
PODXL	0.421	0.0458	0.911	0.0463	0.536	0.00643
SEMA6A	2.7	5.11E-11	2.49	2.30E-05	0.544	0.00671
COL4A5	1.36	0.00393	1.03	0.00757	0.538	0.00678
VWDE	1.56	0.0212	2.14	6.23E-06	0.601	0.0079
QPRT	2.21	0.00013	1.52	0.00774	0.648	0.00851
TMEM56	1.02	0.000101	1.57	3.78E-08	0.546	0.009
DTX4	0.708	0.0257	0.866	0.0465	0.575	0.0092
CDK14	2.48	7.72E-05	1.61	9.36E-06	0.489	0.00995
ADAP2	1.1	0.0191	0.794	0.0409	0.52	0.0103
LINC01559	0.574	0.00574	1.7	0.00437	0.749	0.0105
LRRC8B	0.348	0.0212	0.733	0.0432	0.502	0.0109
MITF	0.777	0.0106	0.596	0.0305	0.512	0.0113
SPNS2	0.607	0.0261	1.95	0.00169	0.543	0.0123
PRDM6	3.02	2.48E-07	1.71	0.000102	0.536	0.0126
GPC4	2.09	8.02E-16	2.25	4.46E-09	0.575	0.0139

CAPN8	2.78	4.64E-06	2.71	6.97E-28	0.516	0.014
MSLN	0.274	0.0334	0.905	0.00784	0.668	0.0152
MARVELD3	2.38	0.0293	2.11	0.0058	0.665	0.0153
LCK	1.54	2.24E-06	0.994	0.0017	0.5	0.0156
PPP1R9A	0.892	0.000576	0.788	0.000327	0.491	0.0163
MARK1	2.52	0.0287	1.39	0.0109	0.512	0.0178
NTN4	1.14	2.00E-04	0.823	0.00129	0.459	0.018
LAD1	0.638	5.77E-08	0.473	0.0284	0.597	0.0184
FAM169A	1	0.000804	1.46	0.00736	0.464	0.0186
LOC202181	1.26	0.000377	0.903	0.00499	0.461	0.0192
PLCB4	1.49	0.013	2.31	0.0015	0.573	0.0193
FUT8	0.636	0.0326	0.813	0.00796	0.613	0.0198
LGR5	1.02	0.0183	1.86	0.00325	0.487	0.0216
PLAC8	0.353	0.0238	0.489	0.0233	0.513	0.022
PCSK6	0.842	4.81E-05	0.566	0.00654	0.445	0.0227
ST14	0.888	0.000461	0.748	0.0425	0.443	0.0228
GALNT18	1.38	3.70E-06	1.24	0.0103	0.473	0.0243
CDH10	4.32	0.0444	2.67	0.00131	0.521	0.0246
ENPP1	0.997	0.0062	1.29	0.0338	0.513	0.0254
PTGFRN	1.01	0.00117	1.05	0.00685	0.477	0.0282
PRICKLE2	2.28	0.00892	2.89	3.39E-10	0.47	0.0289
TNFSF11	1.09	0.0142	1.19	0.0106	0.496	0.0296
SFTA1P	4.08	0.031	5.72	0.0194	0.954	0.0307
SLC16A10	1.54	0.0477	1.23	0.00335	0.438	0.0313
FAM83F	2.34	0.0436	1.89	0.00276	0.466	0.0368
PLA2G12A	0.327	0.0421	0.957	0.00167	0.51	0.0388
FLNC	1.07	0.0117	1.74	0.0327	0.469	0.0389
ELOVL2	3.21	0.0214	1.01	0.0206	0.484	0.0395
BCL11A	2.64	0.0384	3.04	0.00238	0.514	0.0406
WSCD1	1.7	0.0357	1.47	0.0217	0.455	0.0427
HSH2D	0.795	0.0153	0.57	0.0107	0.543	0.0427
DOK5	3.58	0.0143	4.39	0.00117	0.512	0.0454
MGAT3	1.14	0.000198	1.09	4.65E-07	0.446	0.0477
B4GALNT3	1.06	3.25E-05	1.78	7.14E-11	0.396	0.0497
WNT10A	1.45	0.00854	1.78	0.000167	0.545	0.0503
GJB1	0.908	0.0123	2.67	0.0289	0.716	0.0512
UNC13D	0.992	0.0392	1.21	0.0454	0.469	0.0515
LPAR1	0.811	0.0048	0.645	0.0133	0.349	0.058
SLC44A3	0.835	0.000993	0.496	0.0196	0.365	0.0589
OAF	0.279	0.0346	0.852	0.032	0.41	0.0667
GPRC5B	0.661	0.0102	1.47	0.00456	0.446	0.0744
MAP7	1.32	1.79E-07	1.24	3.35E-07	0.359	0.0805
VAV1	2.4	0.000412	2.6	5.35E-10	0.359	0.0827
SYK	2.2	0.00244	1.56	0.000189	0.334	0.091
MARVELD2	0.856	6.89E-08	1.15	0.00022	0.384	0.0933

AGMAT	0.414	0.0471	0.912	0.0105	0.423	0.0964
LRIG1	1.19	0.00306	1.18	0.0358	0.344	0.111
UCA1	0.653	0.00378	1.1	5.57E-05	0.468	0.116
LIPG	0.503	0.0232	1.2	0.00913	0.398	0.136
MPP7	0.618	0.0269	0.723	0.0286	0.353	0.137
TSPAN13	0.849	1.20E-05	0.717	0.0104	0.304	0.17
CLDN9	1.24	0.0488	1.55	0.0286	0.55	0.18
PJA1	0.338	0.00392	0.987	0.00388	0.515	0.247
FBP1	0.374	0.0357	1.17	0.00289	0.281	0.251
DMKN	1.2	1.80E-09	1.75	3.04E-15	0.279	0.337
GPX3	0.564	0.0264	0.763	0.0187	0.271	0.398
EPPK1	1.45	4.41E-05	1.47	0.000923	0.222	0.477
FAM110C	0.528	0.0482	0.972	0.0244	0.222	0.528
NAPRT	0.7	0.0108	1.22	0.011	0.0871	0.801
FOXA2	2.17	3.52E-05	3.24	5.19E-08	0.0301	0.949

Supplementary Table 2

Primers for H2B genotyping

Gene	Forward	Reverse
**HIST1H2BB**	CATAAACCCCACCCCTCAGT	TGTCTTCGCTAACATTCCAGT
**HIST1H2BC**	CACGGATGACAACTGTGCAG	GGCCACAGCTCTTTTAGTGG
**HIST1H2BD**	ACATTGGCATTTGTGACGACA	GGTACTGTGGTGCTAAGTCCA
**HIST1H2BE**	GCATGCAGACTTCACGACAA	GCCTATTTCAGCTGCAGGAG
**HIST1H2BF**	TGCCAGTTTACGATAGGAGC	AGCCTTTGGGATTGGGTATG
**HIST1H2BG**	TGCGTCACTAACACATTGCC	AAACTGGTCTCGATCCGCA
**HIST1H2BH**	GACCTACAAAAACGGCCAGC	GTATTGGCGACACTTCTCCC
**HIST1H2BI**	TAATGAGGGCGTTTGGGCTC	ACAACATGGGTGGCTCTTAG
**HIST1H2BJ**	CTACCAATCAGACACAAGAC	AGGAATACAAGCACCAGCTC
**HIST1H2BK**	GCCAGACTCGATTACAAGCA	TGACCTCTGACGTTACCCTG
**HSIT1H2BL**	GGCATCTGTCGTCATCCACG	GAAGAGCGAACCAGCTCTTC
**HT1H2BM**	GCAAAACTGGCATCTGACGT	ATGTACGAAGCGAGTCCCAA
**HSIT1H2BN**	TGGTTAAACGCACAACTTCATTC	ACCGGGATTAGAGGCTTGAG
**HIST1H2BO**	ACGTCATCTGAGTAACTTCCAA	TTACCTTTTCCCGCCGTCTT
**HIST2H2BE**	ATGAACGACTTTCGGAGCCC	TGATTAGGTGGGTGGCTCTG
**HIST2H2BF**	AGTTAACCAATGAAAGCGCAG	CCTAAACCCGAATGCATCCG
**HIST3H2BB**	GCGTTCGATTGGATGGCTAT	ACCTGAGCAGTTTCTACCCC

Primers for q-RT PCR

Primer name	Forward	Reverse
**5S mRNA**	GGCCATACCACCCTGAACGC	CAGCACCCGGTATTCCCAGG
**GAPDH mRNA**	GACAGTCAGCCGCATCTTCT	TTAAAAGCAGCCCTGGTGAC
**ANXA3-ex2in2**	GGCAAAGGTGGGATATCATGG	CCTTGCTATCTTGACCTTCTGTG
**ANXA3-ex3in3**	TTGGACACCGAGGAACAGTAA	TGTAACTTGCCTGATTTGCTTCA
**ANXA3-ex7in7**	AAGAAGAGTCTTGGAGATGACATT	CATGCAGCTTACTGGGGAGT
**ANXA3-ex12in12**	GGACATTCGAACAGAGTTCAAG	GAGATGGGTAGAGAAGGGCA
**ANXA3 mRNA**	CCTTCGCTCGCAGTTTGTTC	CGGTGTCCAACCCAGATAGA
**SNAP47-ex2in2**	TCTGATTCTCCTTCGCTGTGG	CCTCTCCTGTCGGGAATCG
**SNAP47-ex5in5**	GCAAGAAGATGGAGCTGTTAGA	TTTAGAAGTCTGTGCACGCG
**SNAP47-in6ex7**	CCTCTGCTGTGATCTTGTGC	CATCCAGGGCTTCGTCTTGT
**SNAP47 mRNA**	AGAAGAGCTGCTCAGTCTGGCA	AGTTCCTGGGTATCTGCCTCAG

Primers for ChIP-qPCR

Locus	Forward	Reverse
**ANXA3_+3**	ATACTCAGCTGTCCTCGCTC	CTGTCCCCTAGACCTTTAG

Supplementary Methods

CUT&RUN

Experiments were performed as described in [2] with modifications. Specifically, 5 million S2VP10 H2B knock-in cell lines and parental cell line were incubated with NE1 (20 mM HEPES-KOH, pH 7.9; 10 mM KCl; 0.5 mM Spermidine; 0.1% Triton X-100; 20% Glycerol; proteinase inhibitor cocktail) for 10 min and then with Buffer 1 (20 mM HEPES, pH 7.5; 150 mM NaCl; 2 mM EDTA; 0.5 mM Spermidine; 0.1% BSA; proteinase inhibitor cocktail) for 5 minon ice. Nuclei were collected by centrifugation at 600 g for 3 min, and then resuspended in Buffer 2 (20 mM HEPES, pH 7.5; 150 mM NaCl; 0.5 mM Spermidine; 0.1% BSA; proteinase inhibitor cocktail). 2 μg of specific antibody was added to the nuclei and incubated at 4°C overnight. 4 μl of rabbit anti-mouse IgG (Jackson ImmunoResearch, 315-005-003) was incubated with the nuclei for 1 hour for FLAG CUT&RUN since the anti- FLAG antibody was generated from mouse. After washing the nuclei twice with Buffer 2, 0.5 ng/μl of pA-MNase fusion protein was added to the nuclei and incubated at 4°C for 1 hour. The nuclei was then washed once with Buffer 2 and once with low salt rinse buffer (20 mM HEPES, pH7.5; 0.5 mM spermidine). Nuclei was resuspended in digestion buffer (3.5 mM HEPES, pH7.5; 10 mM $CaCl_2$) and digestion was performed on ice for 15 min. Digestion was quenched by adding 2X STOP buffer containing 144 pg yeast spike-in DNA (40 mM HEPES, pH 7.5; 340 mM NaCl; 40 mM EGTA). Cleaved DNA fragment was released from the insoluble chromatin by incubating at 37°C for 30 min. Supernatant was collected for DNA purification with NucleoSpin Gel and PCR Clean-up Kit (Macherey-Nagel, 740609.250) and libraries were prepared using Ovation Ultralow System V2 (NuGEN, 0344NB). PCR amplification condition was modified to 72°C 2 min, 95°C 3 min, (98°C 20 sec, 65°C 10 sec) for 11-12 cycles, 72°C 1 min. Paired-end sequencing was performed on the Illumina NextSeq 500 Sequencing System.

PRO-seq

5-10 × 10^6 cells were harvested by centrifugation. Briefly, cells were rinsed with ice-cold 1X PBS followed by wash buffer (10 mM Tris-Cl, pH 7.4; 10 mM KCl; 150 mM sucrose; 5 mM $MgCl_2$; 0.5 mM $CaCl_2$; 0.5 mM DTT proteinase inhibitor cocktail; 40 units RNase inhibitor/10 ml (added immediately before use) (Ambion, AM2682) dissolved in nuclease-free water once. Cells were lysed by incubating in lysis buffer (10 mM Tris-Cl, pH 7.4; 10 mM KCl; 250 mM sucrose; 5 mM $MgCl_2$; 1 mM EGTA; 0.05% Tween-20; 0.5 mM DTT; 0.2% NP40; proteinase inhibitor cocktail; 40 units RNase inhibitor/10 ml (Ambion, #AM2682) for 5 min. The permeabilized cells were collected at 4°C, 1000 g for 5 min after two washes with wash buffer and flash frozen in 100 μl storage buffer (50 mM Tris-Cl, pH 8.0; 40% glycerol; 5 mM $MgCl_2$; 0.1 mM EDTA; 0.5 mM DTT). Permeabilized cells in 100 μl storage buffer were mixed with 100 μl pre-warmed 2X nuclear run-on reaction mix (10 mM Tris-Cl, pH 8.0; 5 mM $MgCl_2$; 1 mM DTT; 300 mM KCl; 37.5 μM biotin-11-ATP (Perkin Elmer, NEL544001EA); 37.5 μM biotin-11-CTP (Perkin Elmer, # NEL542001EA); 37.5 μM biotin-11-GTP (Perkin Elmer, NEL545001EA); 37.5 μM biotin-11-UTP (Perkin Elmer, NEL543001EA); 0.8 units/μl RNase Inhibitor (Ambion, AM2682); 1% Sarkosyl (Sigma-Aldrich, # L5125)) and incubated at 37°C for 5 min. The run-on reaction was terminated by adding Trizol LS (Invitrogen, 10296010) and pelleted by ethanol precipitation. RNA pellets were re-dissolved in nuclease-free water and briefly denatured at 65°C followed by base hydrolysis with NaOH to produce 100-150 nt fragments. The biotinylated nascent transcripts were purified three times using Dynabeads™ M-280 Streptavidin (Invitrogen, 11206D), each round followed by Trizol (Invitrogen, 15596026) extraction and ethanol precipitation. The 5' cap of transcripts were removed with RNA 5' Pyrophosphohydrolase (NEB, M0356S) and the 5' hydroxyl group repaired with T4 polynucleotide kinase (NEB, M0201). The libraries were then generated using TruSeq small RNA adapters and size-selected to a range of 140-350bp through Solid Phase Reversible Immobilisation beads (Beckman Coulter AMPURE XP, A63881) before being sequenced using Illumina NextSeq500 with 75 bp paired-end reads.

ATAC-seq

ATAC-seq libraries were generated as described in [3]. 50,000 cells were harvested and washed once with 50 μl of cold 1X PBS and then resuspended in 50 μl of lysis buffer (10 mM Tris-HCl, pH 7.5; 10 mM NaCl; 3 mM $MgCl_2$; 0.1% NP-40). Cells were then centrifuged for 10 min at 500 g. The supernatant which contains the cytoplasmic components was discarded and the pellet was collected. Transp osition was initiated by adding 2X TD buffer with 2.5 μl of Tn5 transposase (Illumina, FC121-1030) in 50 μl total volume. Transposition was allowed to proceed for 30 min at 37°C in a thermomixer shaking at 500 rpm. Transposition reactions were cleaned up with Qiagen MinElute Kit. Libraries were generated using the custom Nextera PCR primers [3]and were amplified for 10–12 cycles. Libraries were purified with AMPure beads to remove primer dimmers and > 1,000 bp DNA. Library quality was assessed using the Agilent

Bioanalyzer High-Sensitivity DNA kit and quantified using the NEBNext Library Quant Kit. Libraries were sequenced on Illumina NextSeq 500 with 50 bp paired-end reads.

ChIP-qPCR

Cells were cross-linked with 1% PFA at room temperature for 5 min and then quenched the formaldehyde with 125 mM glycine at room temperature for 5 min. Cells were washed twice with 1X TBS and harvested by scarping in 1 ml extraction buffer (10 mM Tris-HCl, pH7.5; 10 mM NaCl; 0.5% NP-40; proteinase inhibitor cocktail) and incubated on ice for 30 minutes. Nuclei was washed once with MNase digestion buffer (20 mM Tris-HCl, pH7.5; 15 mM NaCl; 60 mM KCl; 2 mM $CaCl_2$). Digestion was started by adding 5μl MNase (NEB M0247S, diluted 1:10) to the nuclei suspension. The reaction was then incubated at 37°C with 500 rpm shaking for 5 min. Digestion was quenched by adding 2X STOP buffer (100 mM Tris-HCl, pH 8.0; 20 mM EDTA; 200 mM NaCl; 2% Triton X-100; 0.2% sodium deoxycholate). Soluble chromatin was collected after two sequential high-speed centrifugations of the sonicated lysate (10,000 g for 5min and 15 min at 4°C). 5% of the lysate was taken as input and the remaining lysate was incubated with specific antibodies at 4°C for overnight. 30 μl of pre-washed Protein G Sepharose (GE Healthcare, 17061802) were added to each sample and incubated at 4°C for 1-2 hours. The beads were washed with different buffers, once with ChIP lysis buffer, once with lysis buffer with 0.5 M NaCl, once with Tris/LiCl buffer (10 mM Tris, pH 8.0; 0.25 M LiCl; 0.5% NP-40; 0.5% Na-deocycholate; 1 mM EDTA) and twice with Tris/EDTA buffer (50 mM Tris, pH 8.0; 10 mM EDTA). After washing, 100 μl of 10% chelex (Bio-Rad, cat. no. 142-1253) were added to the washed protein-G beads and boiled at 95°C for 10 min and then 5 μl of 20 mg/ml Proteinase K (NEB, P8107S) were added and incubated at 37°C for 30 min. Samples were boiled again for 10 min to inactivate proteinase K and centrifuged to collect the supernatant. 100 μl of 20 mM Tris, pH 8.0 was added to the pellet and centrifuged again to collect the supernatant. The supernatants were combined, and it was used as template for qPCR reaction. qPCR was performed using Applied Biosystems QuantStudio 3 Real-Time PCR System.

Antibodies

Anti- FLAG (Sigma F7425, F1804), Anti-H2A (Immunoway YT2152), Anti-H2B (Cell Signaling Tech 12364S), Anti-H3 (Immunoway YM3038), Anti-H4 (Cell Signaling Tech 13919S), Anti-H2A.Z (Abcam ab4174), Anti-H3.1/3.2 (Millipore ABE154), Anti-H3.3 (Millipore 09-838), Anti-CENPA (Cell Signaling Tech 2186), Anti-H2AK119ub (Cell Signaling Tech 8240s), Anti-H2BK120ub (Cell Signaling Tech 5546s), Anti- Anti-H3K4me3 (Active Motif 61379), Anti-H3K9me3 (Active Motif 61013), Anti-H3K27me3 (Cell Signaling Tech 97335), Anti-H3K36me3 (Immunoway YM3088), Anti-H4K20me3 (Active Motif 39671), H4K16ac (Immunoway YK0014), Anti-NAP1L1 (Santa Cruz sc-81328, Abcam ab33076), Anti-NAP1L2 (Abnova H00004674-D01), Anti-SPT16 (Santa Cruz sc-377028), Anti-SSRP1 (Santa Cruz sc-74536), Anti-HSC70 (Santa Cruz sc-7298), Anti-HIRA (Santa Cruz sc130636), Anti-ATRX (Santa Cruz sc-55584), Anti-DAXX (Abcam ab32140), Anti-B23 (Santa Cruz sc-271737), Anti-C23 (Santa Cruz sc-55486), Anti-CAF1 p150 (Abcam ab126625), Anti-CAF1 p60 (Santa Cruz sc-393662), PCNA (Immunoway YM3031), Anti-α-tubulin (Immunoway YM3115), SPT6 (Santa Cruz sc-393920), HDAC2 (Abcam ab12169), MCM7 (Santa Cruz sc-9966), Anti- 53BP1 (homemade), Anti-γH2Ax (Abcam ab26350), Anti-BrdU (Cell Signaling Tech 52925), H3K9ac (Immunoway YK0006), H3S10ph (Abcam ab5176), H3K27ac (Active Motif 39685), H3K36me2 (Cell Signaling Tech 29015), H3K79me3 (Immunoway YM3091).

CUT&RUN sequencing data analysis

Reads were aligned to human reference genome hg19 and yeast reference genome sacCer3 by Bowtie 2 separately [4]. Human reads were normalized by spike-in yeast reads using deepTools [5]. MACS2 [6] was used to call peaks using parental as control under $p < 0.001$ with paired-end mode. Mutant enriched peaks were identified by 'DESeq2' [7] taking yeast spike-in reads as scale factor after counting reads within peaks using BEDTools ($p < 0.05$ and log2 fold change > 0.5) [8]. Peak annotation to different genomic regions, including gene body, promoter (transcription start site (TSS) +- 1kb), downstream (3kb downstream of transcription end site (TES)) and distal intergenic regions, was performed by R package 'ChIPseeker' [9]. As a random control, the same number of 'shuffled peaks' of the same lengths as mutant enriched peaks were randomly generated for each chromosome for 1,000 times using BEDTools [8]. A Chi-square test was then performed to assess the statistical significance in the difference of genomic distributions between mutant enriched peaks and shuffled peaks. For each gene, the occupancy of FLAG was quantified by the number of reads located in gene body and 1 kb upstream of TSS counted by BEDTools [8], and subsequently normalized by the yeast spike-in factor. To identify genes showing differential occupancy of FLAG between mutants and WT, differential occupancy analysis was further performed by 'DESeq2' ($p < 0.01$ and log2 fold change > 0.5) [7].

RNA-seq data analysis
Reads were mapped to the human reference genome hg19 and counted by STAR 2.6.1a [10] with default parameters. R package 'DESeq2' [7] was used to perform differential expression analysis ($p < 0.05$ and $|\log2$ fold change$| > 0.25$). 'bamCoverage' in deepTools [5] was used to generate bigwig files for IGV visualization using the traditional normalization method: Reads Per Kilobase per Million mapped reads (RPKM). Gene set overrepresentation analysis based on differentially expressed genes was performed by R package 'HTSanalyzeR2' [11] using hypergeometric tests, and significant gene sets were defined by Benjamini-Hochberg adjusted $p < 0.05$.

PRO-seq data analysis
Adapter cutting, reads alignment and coverage files generation were based on the pipeline illustrated by Dig et al. [12] using human genome hg19 as reference genome. Count data was obtained from the bigwig files using R package 'bigWig' [13]. R package 'DESeq2' [7] was used to perform differential expression analysis, and significantly differentially expressed genes were defined by $p < 0.05$ and $|\log2$ fold change$| > 0.25$.

ATAC-seq data analysis
Reads were aligned to the human reference genome hg19 by BWA [14] with default parameters. Reads from mitochondrial were removed and de-duplicated. Only paired reads were used for further analysis. MACS2 was used to call peaks with paired-end mode ($q < 0.05$) [6]. Coverage files for IGV visualization were generated from bam files using deepTools [5].

DRB treatment
5,6-Dichlorobenzimidazole 1-b-D-ribofuranoside (DRB) (Sigma, D1916) was dissolved in DMSO as 75 mM solution stored at –20°C. S2VP10 wild type and G53D mutation cells grew overnight on 35 mm plates to 60%-70% confluency and then were treated with 300 μM DRB for 3.5 hours. Cells were washed with PBS to remove the DRB and then incubated in fresh medium for various time periods. Following the incubation period, cells were washed with PBS and subjected to total RNA isolation using a universal RNA extraction kit (Takara, 9767). 500 ng of total RNA were used for reverse transcriptase reaction according to PrimeScript RT Master Mix (TaKaRa,RR036A). The levels of pre-mRNA at various positions of ANXA3 gene were determined by real-time PCR. Values obtained were normalized relative to the average level of 5S and GAPDH. Results were expressed in relation to the pre-mRNA value of cells treated with DMSO.

Primers
All primers used in this study are listed in Supplementary Table 2.

Data Availability
RNA-seq, PRO-seq, ATAC-seq and CUT&RUN sequencing data sets have been deposited to the Gene Expression Omnibus under accession number GSE134864.

References

1 Kujirai, T. *et al.* Structural basis of the nucleosome transition during RNA polymerase II passage. *Science* **362**, 595-598, doi:10.1126/science.aau9904 (2018).

2 Skene, P. J. & Henikoff, S. An efficient targeted nuclease strategy for high-resolution mapping of DNA binding sites. *Elife* **6**, doi:10.7554/eLife.21856 (2017).

3 Buenrostro, J. D., Giresi, P. G., Zaba, L. C., Chang, H. Y. & Greenleaf, W. J. Transposition of native chromatin for fast and sensitive epigenomic profiling of open chromatin, DNA-binding proteins and nucleosome position. *Nat Methods* **10**, 1213-1218, doi:10.1038/nmeth.2688 (2013).

4 Langmead, B. & Salzberg, S. L. Fast gapped-read alignment with Bowtie 2. *Nat Methods* **9**, 357-359, doi:10.1038/nmeth.1923 (2012).

5 Ramirez, F., Dundar, F., Diehl, S., Gruning, B. A. & Manke, T. deepTools: a flexible platform for exploring deep-sequencing data. *Nucleic Acids Res* **42**, W187-191, doi:10.1093/nar/gku365 (2014).

6 Zhang, Y. *et al.* Model-based analysis of ChIP-Seq (MACS). *Genome biology* **9**, R137 (2008).

7 Love, M. I., Huber, W. & Anders, S. Moderated estimation of fold change and dispersion for RNA-seq data with DESeq2. *Genome biology* **15**, 550 (2014).

8 Quinlan, A. R. & Hall, I. M. BEDTools: a flexible suite of utilities for comparing genomic features. *Bioinformatics* **26**, 841-842, doi:10.1093/bioinformatics/btq033 (2010).

9 Yu, G., Wang, L. G. & He, Q. Y. ChIPseeker: an R/Bioconductor package for ChIP peak annotation, comparison and visualization. *Bioinformatics* **31**, 2382-2383, doi:10.1093/bioinformatics/btv145 (2015).

10 Dobin, A. *et al.* STAR: ultrafast universal RNA-seq aligner. *Bioinformatics* **29**, 15-21 (2013).

11 Gao, F. *et al.* in *Celebrating The 25th Conference on Intelligient Systems for Molecular Biology And The16th European Conference on Computational Biology: ISMB/ECCB 2017.*

12 Mahat, D. B. *et al.* Base-pair-resolution genome-wide mapping of active RNA polymerases using precision nuclear run-on (PRO-seq). *Nature Protocols* **11**, 1455-1476, doi:10.1038/nprot.2016.086 (2016).

13 Kent, W. J., Zweig, A. S., Barber, G., Hinrichs, A. S. & Karolchik, D. BigWig and BigBed: enabling browsing of large distributed datasets. *Bioinformatics* **26**, 2204-2207 (2010).

14 Li, H. & Durbin, R. Fast and accurate short read alignment with Burrows–Wheeler transform. *bioinformatics* **25**, 1754-1760 (2009).
